# Identifying overrepresented concepts in gene lists from literature: a statistical approach based on Poisson mixture model

**DOI:** 10.1186/1471-2105-11-272

**Published:** 2010-05-20

**Authors:** Xin He, Moushumi Sen Sarma, Xu Ling, Brant Chee, Chengxiang Zhai, Bruce Schatz

**Affiliations:** 1 Department of Computer Science, University of Illinois at Urbana-Champaign, Urbana, IL 61801, USA; 2 Institute of Genomic Biology, University of Illinois at Urbana-Champaign, Urbana, IL 61801, USA

## Abstract

**Background:**

Large-scale genomic studies often identify large gene lists, for example, the genes sharing the same expression patterns. The interpretation of these gene lists is generally achieved by extracting concepts overrepresented in the gene lists. This analysis often depends on manual annotation of genes based on controlled vocabularies, in particular, Gene Ontology (GO). However, the annotation of genes is a labor-intensive process; and the vocabularies are generally incomplete, leaving some important biological domains inadequately covered.

**Results:**

We propose a statistical method that uses the primary literature, i.e. free-text, as the source to perform overrepresentation analysis. The method is based on a statistical framework of mixture model and addresses the methodological flaws in several existing programs. We implemented this method within a literature mining system, BeeSpace, taking advantage of its analysis environment and added features that facilitate the interactive analysis of gene sets. Through experimentation with several datasets, we showed that our program can effectively summarize the important conceptual themes of large gene sets, even when traditional GO-based analysis does not yield informative results.

**Conclusions:**

We conclude that the current work will provide biologists with a tool that effectively complements the existing ones for overrepresentation analysis from genomic experiments. Our program, Genelist Analyzer, is freely available at: http://workerbee.igb.uiuc.edu:8080/BeeSpace/Search.jsp

## Background

One of the changes associated with the advances in genomic and systems biology is that biologists are no longer limited to studying one gene at a time. At the conceptual level, this is necessary because functionally-related genes, or modules, create the natural bridge from single genes to the complexity of the entire organism [1]. In practice, biologists study groups of genes found through various ways: for instance, the genes differentially expressed under different conditions in DNA microarray studies; the genes sharing similar expression profiles across a large number of conditions; and the genes showing similar patterns of evolution [2].

An essential component of these studies is the interpretation of a set of genes: whether there is any common functionality among these genes. Typically, this is formulated as the problem of identifying concepts overrepresented in a given list of genes, or simply overrepresentation analysis. This problem is commonly addressed by using Gene Ontology (GO) [3]. In GO system, one gene is associated with a number of terms that are organized in a directed acyclic graph. A statistical test can then be performed to assess the significance of the association of a term with the gene set being analyzed. The standard test for this purpose is hypergeometric test, coupled with some correction to account for multiple hypothesis testing [4-6].

The main limitation of the GO-based methods is their dependence on the existing annotations. The process of annotating genes with some controlled vocabulary requires the efforts of biologist curators, who need to read and digest a large amount of textual information. As a result, it is very difficult to keep up with the rapidly growing literature in almost all biological areas. Furthermore, the curators are limited by the scopes of the existing vocabularies or ontologies. For example, GO coverage of certain biological domains such as diseases and animal behavior, is very inadequate in comparison with its coverage of molecular functions.

The ultimate source of information lies in literature, thus techniques that automatically mine information from text may be able to overcome limitations of overrepresentation analysis based on manually curated ontology. Text mining techniques have found a number of successful applications in the biological domain, for instance, the identification of gene names, and the extraction of protein-protein interactions [7,8]. Text mining has also been applied to analyze gene sets obtained from microarray experiments. A large number of methods attempted to summarize gene functions and reveal gene similarities using text mining. Some of the methods in this category are: the literature profiling method [9], Neighbor Divergence method [10], Latent Semantic Indexing [11], ConceptMaker [12], Non-negative matrix factorization (NMF) [13,14] and Anni [15,16]. The idea is that the terms associated with a gene can be viewed as some kind of text "profile" of this gene, similar to the expression profile measured in microarray experiments. Therefore, the functions and relationship among genes can be explored using the same techniques in expression data analysis, such as cluster analysis for grouping similar genes. This purpose is quite different from ours, which is to explicitly extract overrepresented concepts in gene lists. As such, none of these methods provides a statistical test of significance of concepts, a crucial component of overrepresentation analysis.

It is possible to apply the text mining techniques for the overrepresentation analysis of gene lists. The idea is basically parallel to GO-based analysis: terms co-occurred with many genes in the list are likely to reflect the commonalities of the gene list. GEISHA [17] measures the importance of a term (word or bigram) with respect to a given gene group by its overrepresentation in the document set of this gene group versus some reference group. The TXTGate system [18] creates a "profile" of a gene from the literature about this gene, defining profile as a weighted vector of associated terms. The profiles of all genes in a set will be averaged and the terms with high weights can thus be identified. The program MeSHer [19] extracts MeSH terms from Medline documents of a gene and converts the document set of a gene into a a list of associated MeSH terms. Then the standard Fisher's exact test can be applied to a gene set. The MILANO system [20] could help biologists analyze a gene set by retrieving documents where the gene names and some user-defined terms co-occur. Recently, Leong and Kipling developed a new system, PAKORA, that is an extension of the standard hypergeometric test. PAKORA is motivated by the need of addressing annotation bias, that is, some genes are associated with much more documents than other genes simply because of their biological importance and may bias the statistical tests [21].

The statistical treatment of all these methods are somewhat inadequate. As the authors noted in their paper, the GEISHA method fails to handle the representational bias of genes in literature. The method pools documents from all genes in a group together, as a result, if some gene has a very large number of associated documents, this gene will dominate the document set of this group and thus strongly bias the result. This is certainly a problem as our goal is to uncover common themes in the given gene list, thus we should favor terms that are associated with more genes. In TXTGate, MeSHer and PAKORA, the important information of how often a term co-occurs with a gene is ignored. Instead, if a term co-occurs with a gene, it will be considered as an association and will be used for the statistical test of overrepresentation. This is highly undesirable because co-occurrence alone is not a very reliable indicator of semantics, so it is important to attach some confidence value to the association between terms and genes. There is some other statistical bias in the TXTGate approach of averaging gene profiles, which is explained in [15]. MILANO is mainly a system for retrieving and navigating articles about gene sets, but does not provide any statistical test for important terms.

In this paper, we proposed a new method based on a rigorous statistical model to identify overrepresented concepts in gene lists from free-text. We implemented a system that allows users to perform the proposed analysis efficiently. Furthermore, our program is embedded into BeeSpace http://www.beespace.uiuc.edu/, an integrated environment for biomedical literature retrieval and mining, to take advantage of its many features. We evaluated our program, Genelist Analyzer (or simply Analyzer), on several gene lists, including one simple list commonly used for evaluation and two large lists derived from our microarry experiments of social behavior of honey bees. We compared our results with the standard GO term enrichment analysis and found that Analyzer is capable of recovering the important themes in GO analysis and providing additional information when GO analysis is not very informative. In addition, we compared Analyzer with two other methods that are closest to ours, GEISHA and PAKORA, and demonstrated that our method is statistically superior and offers additional features that are beneficial in practice.

## Results

### Genelist Analyzer: a system for identifying important concepts in gene lists

**Figure 1:**
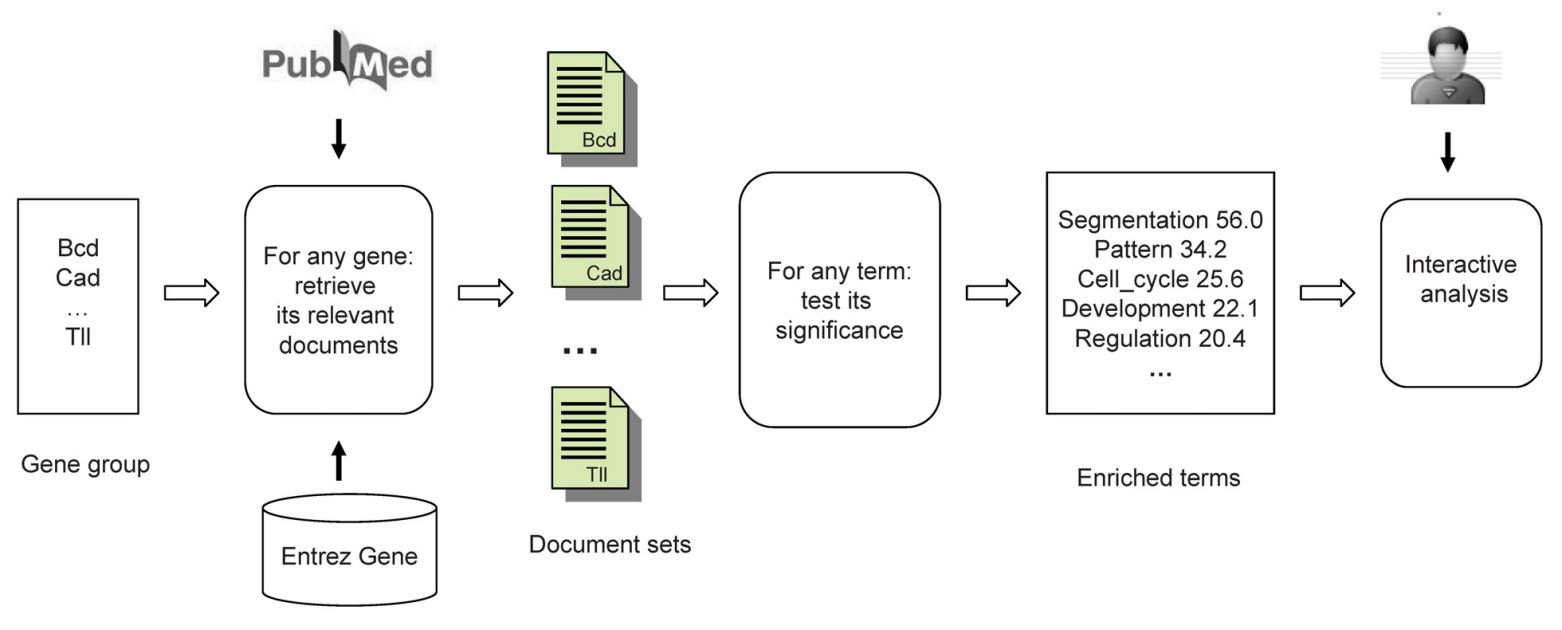
**Conceptual overview of Genelist Analyzer**. The program takes a group of genes as input, retrieves the relevant documents for each gene, and identifies the terms that are associated with this gene group (enriched terms). Further interactive analysis will allow a user to trace back the documents containing the terms and genes. The example shown in this figure is hypothetical.

We developed a system, Genelist Analyzer, for experimental biologists to conduct overrepresentation analysis on gene lists. The program, Genelist Analyzer, can be accessed at: http://workerbee.igb.uiuc.edu:8080/BeeSpace/Search.jsp. Our system uses the textual terms (words and phrases) from literature, thus overcoming the limitations of controlled vocabularies such as GO. A conceptual overview of our system is shown in Figure 1. We created organism-specific document collection from Medline. The retrieval of documents for a given gene is based on the mapping from gene identifiers to synonyms, as curated in the Entrez Gene database [22]. Our novel statistical method is used to identify terms that are most likely to represent the common features of the input gene list. The program outputs the most enriched terms and their significance values (LRT scores). Further support is provided to allow users to examine the gene-term associations and navigate the literature. The details of how each step is implemented are described in Methods.

**Figure 2:**
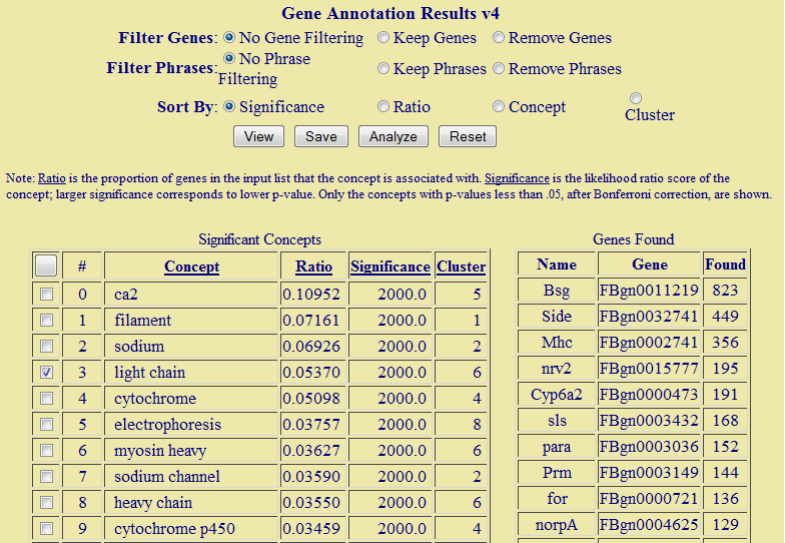
**The result page of Genelist Analyzer**. The top part of the screen provides control of the output; the "Significant Concepts" table displays the main concepts identified along with the relevant statistics (note that gene names are underline and linked to the external program Gene Summarizer). The Ratio field of a concept is the percentage of genes associated with this concept and the Significance field displays the statistical confidence score of that concept. The concepts are automatically clustered, and the index of the cluster which a concept belongs to is also shown. The "Genes Found" table display the information of the input gene: the name and the number of documents retrieved for each gene. This page is generated from the genes up-regulated by methoprene treatment in honey bees.

**Figure 3:**
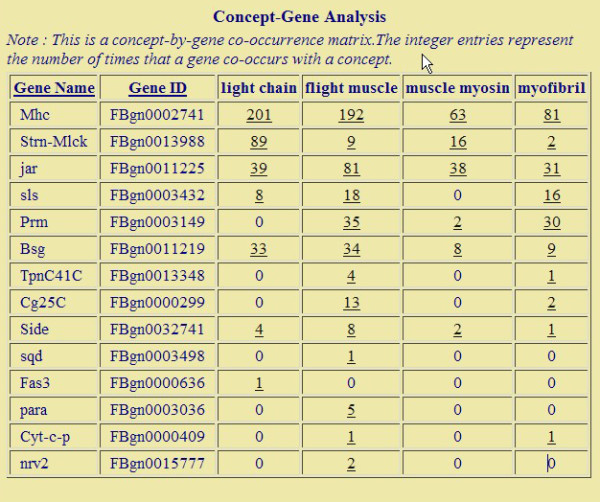
**The gene-term matrix from Genelist Analyzer**. When the user chooses specific concepts of interest from the result screen and click the "Analyze" button, the program will retrieve the genes that actually contain these concepts, ranked by their relevances, and display the gene-term matrix. The supporting documents of gene-term association can be accessed through the hyperlinks in the matrix. This page used the list of genes up-regulated by methoprene treatment in honey bees.

Below, we briefly describe the general analysis procedure with our system (the detailed information of how to run the system can be found in the website). In the Analyzer interface, a user could choose an organism, a document collection (which may be created by the user as explained above), and paste the gene list he wants to investigate. The result page lists the most overrepresented terms, their LRT scores and the estimated percentage of genes related to the terms (Figure 2). If a term is recognized as a gene name, it will be highlighted and linked to the Gene Summarizer program in BeeSpace [23], which is able to automatically generate summaries of genes from literature text, covering aspects such as gene expression patterns and genetic interactions. The user has a flexible control of the results: he could choose what to display (genes or ordinary terms; words or phrases) and how to sort them. Analzyer allows a user to select terms of interest and explore the genes related to the selected terms. This is accomplished by first selecting some terms of interest in the result page, and then analyzing the gene-term association matrix (this is trigged by clicking the "Analyze" button after terms are chosen). The matrix will show the counts of chosen terms in the documents of each gene (Figure 3). In particular, the genes are ranked by their relevance to the user-selected terms, using the standard TF-IDF scoring method in information retrieval. If the input gene list is large and many significant terms are identified, this gene-term matrix will be a very easy way for one to focus on a subset of genes and terms. If the count for a gene-term pair is not zero, the user can follow the link and will be redirected to the documents that mention both the term and the gene. This stands in contrast with GO-based analysis, where the lists of overrepresented terms are the end results. It is not straightforward to retrieve documents about GO terms as typically they do not appear in text in their exact forms. In our system, the exploration of documents could serve as a starting point for further analysis. We also note that for every concept, our program will estimate the proportion of genes in the input that are associated with this concept, the weight of the foreground distribution under our mixture model (the parameter *θ*). Thus to further guard against the representational bias, a user could simply ignore the concepts that are statistical significant, but have low weights (called Ratio in the output screen, see Figure 2). Finally, we note that Analyzer is highly efficient. It takes less than three minutes to execute a query consisting of 173 genes in the document collection of fruit fly, and small queries typically take less than a minute.

### Analyzer addresses the representational bias

We show that Analyzer solves the representational bias problem by comparison with GEISHA. The problem is defined as the bias of results toward terms from well-studied genes which tend to have a large supporting literature and dominate the relevant document set. It is easy to appreciate the importance of this problem when doing text-based gene list analysis: for example, the query of gene "p53" returns 43,200 abstracts in PubMed search; while a large proportion of genes in all model organisms have no associated abstracts.

We evaluated our program on the gene cluster K from the yeast microarray data of cell-cycle in [24]. This is also one example used in [17]. In this cluster of 15 genes, 12 are genes in respiratory complex, one (VDAC, voltage-dependent anion channel) is a membrane channel and the last two are a metabolic enzymes. In their paper, the authors of GEISHA noted that VDAC was an extensively-studied gene and dominated the results. A large portion of the top terms were those about VDAC, including: "voltage-dependent", "pores", and "channel" [17]. Our experimentation confirms the representational bias: VDAC returns 59 documents in the yeast collection, while the second most represented gene returns only 19 documents. However, none of the above VDAC associated terms appear in the top 100 terms identified by our program. Our analysis, instead, produces many terms related to respiration in the top 50 terms, such as: "electron transport", "respiratory", "mitochondrial membrane", "oxidoreductase", "succinate dehydrogenase", "ubiquinone", "cytochrome", etc (the genes and the top 50 terms are listed in Additional file 1 Table S1). Other terms (not shown here due to space limitations) are often related to respiration, sometimes indirectly, and many of them are names of related genes. Based on this example, we observe that our program does not suffer from the gene representational bias problem that affects the performance of a text-mining program for gene lists, if not handled properly.

One possible concern with the overrepresentation analysis is that it may identify false conceptual themes simply by chance. It is generally difficult to quantify the precision of this analysis as whether a concept is related to a gene is somewhat subjective, so we instead analyze a list of genes randomly sampled from yeast genome (see Additional file 1 Table S2) and see if the results of Analyzer contain misleading themes. Interestingly, we did find a set of concepts possibly related to heat shock, such as "hsp gene" and "stress response". Further inspecting the gene-concept matrix allows us to identify the related genes, Hsp26, Cne1, Uga2, Stu1 and Rtg3 (Additional file 1 Table S3). Indeed, both Hsp26 and Cne1 are chaperons that respond to heat shock, and other genes are all related in some way. For instance, we find two articles using our system that supports the relationship between Rtg3 and stress response [25,26]. The results are in fact not surprising, retrospectively, given that hundreds of genes are related to stress response in yeast [27]. Since no other conceptual themes were identified by Analyzer besides the stress-related one, it seems that Analyzer does not provide false, misleading findings. Instead, Analyzer was able to suggest subtle and unexpected concepts that are biologically meaningful in this example.

### Comparison with GO-based enrichment analysis on a honey bee gene list

We next analyzed the gene lists derived microarray experiments on honey bee social behavior. In a honey bee colony, the worker bees carry out different tasks for the colony depending on their age. This age dependent performance of tasks is called behavioral maturation. Younger bees stay in the hive and take care of feeding the larvae, these workers are called nurses. Once they get older, they transition to collecting food outside the colony and are called foragers. Genome-wide analyses of brain gene expression using microarrays have been carried out to understand the molecular events that accompany behavioral maturation in the Western honey bee *Apis mellifera *and other species [28,29]. We performed analysis of genes identified in these datasets with the Analyzer in order to evaluate its performance, and compared the results with those from GO-based analysis and from other text-mining systems. We use the *Drosophila *orthologs of honey bee genes, as the annotation (in terms of GO) and the literature about the fly orthologous genes are the standard information used by bee biologists most of the time. Similarly we used the *Drosophila *document collection as our background. The particular implementation of GO-based analysis we chose is GOToolBox [5].

In the first experiment, we analyzed the list of genes that showed species differences in regulation during behavioral maturation [29]. Table 1 shows the comparison between Biological Process terms that were found to be enriched by GOToolBox and the first 100 terms retrieved by Genelist Analyzer. We manually group these terms based on relatedness and/or commonality of genes underlying these terms and show the related terms from the Analyzer results. Expert biologists who are the intended users of Analyzer would be able to do this easily. We see that most of the GO terms had corresponding terms retrieved by Analyzer. This is an important validation of Analyzer, as it is able to recover the important conceptual themes from plain text without involving manual annotation of genes. The Analyzer also retrieved terms that did not have corresponding matches in GO because GO lacks enough behavior annotations. The most notable of these was the term diapause, which is the term for insect hibernation during winter. An analysis of this term and the associated genes revealed abstracts that show heatshock proteins being regulated in diapausing insects [30,31].

**Table 1:** Overrepresented concepts in bee behavior-related genes identified by GO Toolbox and Genelist Analyzer.

GO Toolbox	Genelist Analyzer
Defense response	Defense, cytokine, *fkbp52*, *cactus*, fibroblast
Response to stress, response to heat, response to temperature stimulus	Thermotolerance, *Hsp *(heatshock protein), *hsf, hs, droj1, hsp40, hsp68, hsp23, hsp26, csp, trap1*
Protein folding	Chaperone, cochaperone
Pigmentation, Dopamine metabolism, Catecholamine metabolism	Pigment, melanin, Laminin
Carbohydrate metabolism	Proteoglycan
Regulation of circadian rhythm	
Circadian sleep/wake cycle, sleep	
Transition metal ion homeostasis, Iron ion homeostasis	Ire, ferritin
Amino acid and derivative metabolism	Alanine
Sex determination	*Pap*
Response to pest, pathogen or parasite	Bacteria, bacterial, gram, pathogen, macrophage, antimicrobial, imd

### Analyzer provides novel insights on a honey bee gene list

In our second example of honey bee gene analysis, we will illustrate a strength of Analyzer in bringing out associations that GO enrichment analysis could miss. Treatment of nurses with hormone analogs like methoprene and chemicals such as manganese and cGMP results in precocious foraging [28]. We next applied Analyzer on the set of genes up-regulated in the brain by methoprene treatment. This set has 166 genes out of which 69 have orthologs in *Drosophila melangoster *and match at least one document in Medline. GO-based analysis for this gene set did not suggest any statistically significant terms. In contrast, Analyzer identified a few hundred significant terms. We present the top 30 terms in Table 2 (according to the order of decreasing significance). Manual inspection of these terms immediately revealed a myosin-related theme: light chain, myosin heavy, heavy chain, thick filament, flight muscle, myosin light, muscle myosin and myofibril (note that for the phrases, myosin heavy and myosin light, the term, chain, is missing due to the fact that we only use bigrams for phrases). Inspection beyond the top 30 terms revealed more terms in this category: nonmuscle (32), mhc gene (34), drosophila myosin (41), nonmuscle myosin (55), myosin ii (56), flightin (57), sarcomere (58) (the numbers indicates ranks of terms. The rest of terms are not listed here).

**Table 2:** Overrepresented concepts in genes responding to methoprene treatment, identified by Genelist Analyzer (top 30 terms) and PAKORA (at *P *< 0.01).

Genelist Analyzer	Ca2, filament, sodium, light chain, cytochrome, electrophoresis, myosin heavy, sodium channel, heavy chain, cytochrome p450, polyacrylamide gel, thick filament, flight muscle, Na channel, myosin light, pyrethroid, channel gene, indirect flight, basement, basement membrane, kdr, proteasome, chain kinase, tubule, insecticide, iv, ATPase, muscle myosin, myofibril, dh31, indirect
PAKORA	phototactic, type, myosin, depressor, lattice, rod, insoluble, separation, resistant, oscillatory, flight, overlap, would, atpase, well, myofibril, built, sarcomere, time, rearing, corresponding, smooth, wall, there, ethyl, disappear, five

To further explore the significance of this semantic theme, we extracted genes related to four chosen terms using our system. This generated a gene-term association matrix (Figure 3). We verified that the top five genes are indeed related to myosin: *Mhc *(myosin heavy chain) and *Prm *are part of myosin complex, *Strn-Mlck*, *jar *and *sls *bind to myosin light chain or regulate its activity. http://www.flybase.org. Myosin molecules form intracellular molecular motors that are part of the cytoskeleton. Molecular motors are known to be important for neural development [32], through processes such as cellular migration and transport of molecules within the cell [33]. This is significant because it is well known that the transition from nursing behavior to foraging behavior is accompanied by structural changes in the brain [34], which may involve these processes. It is likely that methoprene treatment triggers changes in the brain that accompany the behavioral transition from nursing to foraging. Thus Genelist Analyzer performed better than GO analysis in this example by uncovering an interesting biological insight that could be used to enhance our understanding of molecular underpinnings of behavioral maturation.

### Comparison with other text mining tools

Having demonstrated that text mining can complement GO-based analysis, we want to test if other text mining systems for overrepresentation analysis are able to provide similar benefits. Since GEISHA is only implemented for E. coli and yeast [17], we tested the program that is closest to our goal and methodology, PAKORA [21]. No significant terms can be identified for the second honey bee gene list (genes up-regulated in the brain by methoprene treatment), when either Bonferroni correction (the default option) or FDR is used to adjust the *p *values. We thus use 0.01 as the cutoff for raw *p *values (given that thousands of terms are tested simultaneously, this is a very loose threshold. Indeed, the threshold used by Analyzer is much stronger). This leads to 27 overrepresented words shown in Table 2. We found that the terms generated by PAKORA are much less specific, for example, top results include words such as, type, well and five. There are three myosin related words: myosin, myobibril and sacromere. However, given this much smaller list of terms and a much looser threshold of p values, it is unclear whether this theme is really relevant to the query gene list. The main reason for the difference between Analyzer and PAKORA in their abilities of detecting statistically significant terms, we suspect, is that the word count information is ignored in PAKORA. The association between a word and a gene is binary, instead of numerical, as done in our method. The statistical power is likely to be significantly reduced. Two other differences exist between PAKORA and Genelist Analyzer. First, PAKORA is completely based on words while Analzyer allows phrases. We found that this makes an important practical difference: many informative terms are phrases, for instance, light chain, nonmuscle myosin. Second, PAKORA does not offer a simple way to get back the subset of genes related to interested terms. We found that without this feature, it is difficult to interpret the results. In this example, being able to identify five genes related to the myosin theme is crucial for us to validate our findings and points out a way of further analysis (exploring the roles of these genes in behavior maturation).

Next, we compare Analyzer with another text mining tool recently developed, SENT [14]. SENT can also be used for analyzing gene lists from literature, though based on a somewhat different philosophy from Analyzer and PAKORA. Simply speaking, the gene-term matrix is constructed from the literature text according to the co-occurrence between genes and terms. Then genes are grouped according to the similarity of the terms they are associated with (textual profile of genes), and for each group, the most relevant terms are identified as the "semantic feature" of that group. Technically, gene grouping and semantic feature identification are performed via non-negative matrix factorization (NMF). The general idea of grouping genes based on their literature profiles has been implemented by several computational tools for gene list analysis and we think SENT is a representative of these methods. We note that our goal is finding the overrepresented concepts in a gene list, regardless of how genes are grouped, and is a direct extension of the commonly used GO-based enrichment analysis. SENT and other tools such as Anni are not designed for this purpose. Nevertheless, we will perform some comparative analysis to assess the relative strength and weakness of the two strategies.

Since SENT does not support fruit fly, we used the same yeast gene list discussed before as the test case. We ran SENT on this list of 15 genes, setting the number of factors at five (following the guideline of SENT). The resulting five gene groups and their characteristic terms are listed in Additional file 1 Table S4. We note that while Analyzer detects the major theme of electron transport chain and respiration (12 out of 15 genes are related to this theme, see above), SENT results have a much more refined structure. For example, Group 3 consists of four subunits of the enzyme succinate dehydrogenase (Sdh1/2/3/4), and Group 1 contains only a singe gene Ach1, which has a somewhat different function from other genes. By doing so, however, the semantic theme that connects the majority of genes in the list may become less obvious. For instance, even though succinate dehydrogenase participates in electron transport chain, the semantic feature of Group 3 does not have this or closely-related concept; instead, the characteristic terms are those that capture specific details of succinate dehydrogenase (instead of its broader function). We suspect that this may be a general feature of the clustering-based strategy for gene list analysis: the methods are tuned for finding the internal structure of the gene list, but not for revealing the common conceptual theme unifying the genes. At the practical side, we note that SENT does not offer a simple way to examine the relationship between genes and specific concepts of interest. For example, the semantic feature of Group 5 contains a term "life span" and a user may be interested in exploring exactly which genes are related to this concept and what are the supporting documents. However, SENT does not show specific genes related to a user-selected concept. And the documents it extracts for a group is based on the relevance of a document to the semantic feature of the entire group, which in this case is dominated by terms such as NADH, mitochondria, that are only remotely related to life span. In summary, we find that SENT is optimized for the task of gene clustering, while Analyzer is more likely to identify important concepts capturing the commonality of genes, and is more flexible in supporting the exploration of gene-concept relationship.

## Discussion and Conclusion

In this paper, we present a new method for automatically extracting conceptual themes, in the form of overrepresented terms, in a set of genes from literature text. Compared with existing methods for annotating gene lists, our system provides several important benefits: (1) Our analysis is based on free text, using both words and phrases as conceptual units, thus overcomes the limitation of fixed ontology. (2) We developed a novel statistical method that is more rigorous and robust than earlier approaches. (3) Our system supports the interactive analysis of retrieving genes from a subset of terms and literature navigation. (4) By linking with the integrative system, BeeSpace, we offer unique software services, most notably, the customization of document collections and text summarization of genes through Gene Summarizer. In our experimentation, we found that our method could reasonably summarize the literature information of gene lists, sometimes providing useful information missing from the standard GO-based enrichment analysis. Our comparison with other text mining systems with similar purpose showed that our system is statistically more rigorous, and offers more useful features in practice. We anticipate that our system will be particularly useful for two situations: when the analysis based on fixed ontology is uninformative because of the lack of coverage of the ontology; and when a user needs to perform more in-depth investigation of the primary literature, because in general, the ontology may not be directly associated to the literature text, as is the case for GO.

One main issue with our current system is the procedure of retrieving documents for genes. Our method is based on simple string matching of text words and gene names. The possible ambiguity of gene names (e.g. in fruit fly, a number of gene names are English words such as white, for) is not explicitly handled. This does not seem to be a very serious problem for detecting overrepresented concepts, where the results are based on statistical patterns, thus may be relatively insensitive to individual cases of ambiguity. However, we found that name ambiguity does affect the downstream analysis of extracting genes related to terms and the associated literature. Since gene name ambiguity is a general problem affecting many literature mining tasks, one of our ongoing projects is to develop a disambiguation method as part of the BeeSpace infrastructure [35].

Another issue with the current system is the presence of a significant number of non-informative terms often in the results, even if stringent threshold is used for choosing terms. This seems to be an inherent weakness of text-mining analysis, comparing with GO-based analysis. By testing all possible terms in free text, we avoid the constraint posed by defined ontologies such as GO, but also introduce many potential non-informative terms and some of which may happen to be statistically significant. Indeed, we noticed that the other tools we tested, PAKORA and SENT, have the same problem (Table 2 and Additional file 1 Table S4). There does not seem to be a simple solution of this problem without sacrificing the completeness of literature text (as opposed to a limited ontology). In practice, we find this sometimes inconvenient, but does not remove the main patterns in the results. Most often, a biologist user would look for a set of conceptually coherent terms, as we did in this work, and this conceptual theme, as a whole, is a much more robust signal of semantic relationship of genes.

There are a few remaining issues. Firstly, our procedure of document retrieval for genes is quite simple (see, so may lead to unstable performance (when a gene list happens to have many ambiguous genes). We are currently developing a disambiguation method for gene names extending the published work [35]. Secondly, tagging semantic categories of terms identified by Annotator would make the results easier to read and analyze, for example, a user could choose to only look at terms related to a certain aspect thus eliminating many significant but uninteresting terms. Possibilities are MeSH and ontologies maintained at http://obofoundry.org/. Finally, we will work closely with biologists to test the effectiveness of our system for real-life discoveries. After all, to aid the interpretation of results from genomic experiments for biologists is our ultimate goal.

We discussed the relevance of the representation bias of genes in literature to our problem, and our comparison with an existing method suggested that our method is free from the effect of this bias, at least to a large extent. We did not directly compare our method with other recent systems including TXTGate, MeSHer and Anni, because some advantages of our method are obvious, such as weighting the literature evidence of gene-term association; and because these systems use a controlled vocabulary (often MeSH) instead of general words and phrases. We pointed out though, our statistical methodology is independent of the choice of terms.

## Methods

**Figure 4:**
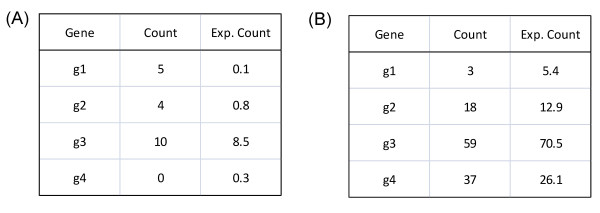
**Simple examples for the term significance test**. Each table represents the (hypothetic) data for one test term. The second column shows the count of the test term in the document set of a gene, and the third column shows the expected count according to the null distribution (assuming that the term is not related to the gene). The expected count is the product of the frequency of the term in the background collection and the length of the document set of the gene. E.g. in the first row of table (A), 5 means the term appears five times in all the documents associated to g1, and 0.1 is the expected counts according to the background. (A) An example where the term may be related to the first two genes. (B) An example where the term does not appear to be significantly related to any gene.

We start with some intuitions of our model. We assume each gene is associated with a set of documents, which we will call the document set of this gene (thus, there is one-to-one correspondence between a gene and a document set). The problem of assigning documents to genes will be discussed in the next section. Our goal is to find all terms that capture the commonalities of the gene set. We test each term independently, and for any term, we look at how often it occurs in the document sets. Figure 4 illustrates the data we have for testing significance of a term (two example terms are shown): the second column shows the count of the term in each document set. In general, some genes will be truly relevant to this term, in the semantic sense; while other genes are not relevant, but may accidentally contain this term in their document sets. The counts of this term in the relevant genes will be high while its counts in the non-relevant genes will be low but not necessarily zero. The term count alone, however, is not very informative, as a term may occur many times by chance if the document set is large enough, so the term count has to be evaluated against the expected counts from chance occurrences (the third column of Figure 4). For the purpose of testing significance of a term, we need a single statistic to summarize the data in the form of Figure 4, that evaluates whether there are a large number of genes where the term counts are significantly higher than expected by chance. For instance, we may infer that the term in Figure 4A is significant as two genes appear to be associated with this term (the counts in the first two genes appear much higher than the expected values), and the term in Figure 4B is probably not significant as none of the genes appears to be associated with the term. The statistical task can be accomplished by using a finite mixture model, where the count of a term follows one of the two distributions: its rate of occurrence is higher in the relevant document sets and lower in the non-relevant document sets. In our case, we use Poisson distribution for term counts, which has been used in previous studies for modeling word distribution [36], and the difference between the two distributions is the rate of the Poisson distribution.

Next we describe our statistical procedure of testing the significance of one term, and the same procedure can be applied to any number of terms. Specifically, we evaluate the significance of a term *t *with respect to a list of *n *genes (thus *n *document sets). Let *d*_1_, *d*_2_, ..., *d*_*n *_be the size of the document sets of the *n *genes, respectively; and *x*_1_, ..., *x*_*n *_be the counts of *t *in the *n *document sets. Let *θ *represent the proportion of the genes relevant to the term *t*, and *λ *and *λ*_0 _be the rates of *t*, under Poisson distribution, in its relevant and non-relevant document sets respectively (note that the values of these parameters may be different for different terms). We assume that the observed data *x*_1_, ... *x*_*n *_are generated by the following process: for each *i*, 1 ≤ *i *≤ *n*, first sample a variable *z*_*i *_from Bernoulli distribution of parameter *θ*, *z*_*i *_= 1 suggests this gene is relevant to *t*, *z*_*i *_= 0 suggests otherwise. Then *x*_*i *_is sampled from Poisson distribution of mean *λd*_*i *_if *z*_*i *_= 1; or sampled from Poisson distribution of mean *λ*_0_*d*_*i *_if *z*_*i *_= 0.

The values of the parameter *λ*_0 _for a term *t *can be approximated by the frequency of this term in the entire collection (called background collection). The parameters *θ *and *λ *can be estimated by the maximum-likelihood method. The likelihood function is given by:(1)

where Pois(·) is the probability density function of the Poisson distribution. We use the standard Expectation-Maximization (EM) algorithm to maximize this function [37]. The formulas for updating *θ *and *λ *are given by:(2)(3)

where *w*_*i *_is the posterior probability of *z*_*i *_= 1 given the current estimate of parameters:(4)

The details of deriving the update formulas are described in the Supplementary Materials.

The statistical significance of the term *t *is evaluated by the standard likelihood ratio test [37]. The null hypothesis is that *t *is not relevant to any gene, which is equivalent to say, *θ *= 0; and the alternative hypothesis is *θ *> 0. The test statistic is expressed as:(5)

where  and  are maximum likelihood estimator (MLE) of *θ *and *λ *respectively. The asymptotic distribution of *T *is known to be χ^2 ^distribution with degree of freedom equal to 2 in our problem. While it is known that this asymptotic distribution is not strictly followed in testing mixture models because of singularity (*θ *= 0 corresponds to the boundary of the region of the parameter values), the real distribution is actually very similar to χ^2 ^[38]. To test multiple terms simultaneously, we applied the Bonferroni Correction.

We explain here why the proposed method is statistically superior to the other methods. First, it does not suffer from the problem of representational bias of genes in literature. Suppose some gene is particularly well-studied and has a much larger set of documents than the other ones. In the GEISHA method, the documents from all genes are pooled, thus the frequency of a term only relevant to the less studied genes in the pooled document set must be low and will not be considered significant. In our model, such terms will be still significant as long as the number of co-occurrences with the less studied genes significantly deviate from the null model. Secondly, our method does not make the assumption that if a term co-occurs with a gene, then they must be associated, as TXTGate, MeSHer and PAKORA do [18,19,21]. Rather, how significant the co-occurrence count is will depend on the term frequency in the background collection and the size of the documents being examined. Finally, the approximate proportion of genes that are truly relevant to a candidate term is estimated (*θ*). This will allow a user to set additional criterion for choosing terms to inspect, for example, only those that capture a minimum percentage of genes in the input set.

### System implementation

The entire Medline collection (abstracts only, no full text) was indexed using the Indri toolkit http://www.lemurproject.org/indri/. We applied a customized program to tokenize the text, which aims to normalize and preserve the integrity of biological entities. These include a number of rules, some typical ones are: the hyphen symbol is removed if it appears between a word and a digit (e.g. brca-1 will be converted to brca1) and replaced by a space symbol if between two consecutive words (e.g. down-regulate will be converted to "down regulate"). We created a few organism-specific document collections, which are used for retrieving gene-related documents. These include collections for yeast, fruit fly and mouse (102447, 38844 and 856833 abstracts, respectively). These collections are created by querying the Medline collection with species names, "yeast", "Drosophila" and "mouse", respectively. We note that a user is allowed to create his own document collection using the BeeSpace infrastructure. For example, a user may choose a collection about insect behavior, and the enriched terms identified in this collection will be more targeted in the behavior domain. This facility of adding collections within BeeSpace enhances the utility of Genelist Analyzer, since these can be used as problem-specific backgrounds. The terms in our analysis include both words and phrases (bigrams). Phrases are extracted from the document collections by the package NSP, Ngram Statistics Package [39]. We used the χ^2 ^test to rank the bigrams and selected the top 20 k bigrams for each collection. Stop words and common English words are removed from the term lists.

For retrieving documents of given genes, we downloaded the Entrez Gene data for gene information [22].

We preprocessed the synonym lists in the downloaded raw data, roughly following the procedure in ProMiner [40]. The main purpose is to remove uninformative and often ambiguous names such as "none" and add some lexical variants of names. The input genes should be specified as gene identifiers, defined by the model organism databases, e.g. for *Drosophila *genes, these should be FLYBASE ids. An input gene id will be mapped to all its synonyms using Entrez Gene data and documents matching any of the synonyms in the corresponding organism-specific collection will be retrieved. Note that Entrez Gene was only used for finding all possible synonyms of a gene identifier, and we did not use its other resources such as GeneRIF.

## Authors' contributions

XH, CZ and BS designed the study. XH implemented the program with the help from XL, BC and other BeeSpace staffs. XH and MS performed the analysis of gene lists using the system. XH, MS and BS wrote the manuscript. All authors read and approved the final manuscript.

## Supplementary Material

Additional file 1 **Supplementary materials**. This file contains additional information of the statistical inference procedure: the estimation of parameters under the EM algorithm. It also contains additional results cited in the main text. Table S1. the results by Genelist Analyzer for 15 yeast genes clustered in microarray experiments. Table S2. a list of genes randomly sampled from the genome of S. cerevisiae. Table S3. the results of Genelist Analyzer for the random gene list Table S2. Table S4. the results of the program SENT from the analysis of the yeast gene list used in Table S1. Click here for file
