# Supplementary material for: Identifying overrepresented concepts in gene lists from literature: a statistical approach based on Poisson mixture model
Source: BMC Bioinformatics. 2010 May 20;11:272. doi: 10.1186/1471-2105-11-272 (PMC2885378; doi:10.1186/1471-2105-11-272)
Supplement: Additional file 1 — Supplementary materials. This file contains additional information of the statistical inference procedure: the estimation of parameters under the EM algorithm. It also contains additional results cited in the main text. Table S1. the results by Genelist Analyzer for 15 yeast genes clustered in microarray experiments. Table S2. a list of genes randomly sampled from the genome of S. cerevisiae. Table S3. the results of Genelist Analyzer for the random gene list Table S2. Table S4. the results of the program SENT from the analysis of the yeast gene list used in Table S1. [file 1471-2105-11-272-S1.PDF]

SUPPLEMENTARY METHODS

Parameter estimation by the EM algorithm

We use the maximum likelihood (ML) method to estimate the parameters $\Theta = (\theta, \lambda)$ for each term to be tested. The objective function is given by Equation (1) in the text. Maximization of this likelihood function can be performed with the standard EM algorithm, where the hidden variables are $Z_i, 1 \le i \le n$, indicating whether the *i*-th gene is related to the term (there are a total of n genes). At the E-step, we compute the expected value of the log-likelihood function with respect to the condition distribution of Z given the observed data under the current estimate of the parameters, $\Theta^t = (\theta^t, \lambda^t)$:

$$
\begin{aligned}
Q(\Theta \mid \Theta^t) &= E_{Z|x,\Theta^t} \log P(x, Z \mid \Theta) \\
&= \sum_{i=1}^{n} [P(Z_i = 1 \mid x_i, \Theta^t) \log P(x_i, Z_i = 1 \mid \Theta) + P(Z_i = 0 \mid x_i, \Theta^t) \log P(x_i, Z_i = 0 \mid \Theta)] \qquad \text{(S1)} \\
&= \sum_{i=1}^{n} \{w_i [\log \theta + \log Poisson(x_i \mid \lambda d_i)] + (1 - w_i)[\log(1-\theta) + \log Poisson(x_i \mid \lambda_0 d_i)]\}
\end{aligned}
$$

Note that we use w_i to represent the conditional probability $P(Z_i = 1 \mid x_i, \Theta^t)$, following Equation (4) in the text. At the M-step, to maximize Q, we take the partial derivative with respective to θ and λ, respectively. Plug in the function for Poisson distribution, we obtain the following equations:

$$\frac{\partial Q}{\partial \theta} = \sum_{i=1}^{n} [w_i \cdot \frac{1}{\theta} - (1 - w_i) \cdot \frac{1}{1-\theta}] = 0 \qquad \text{(S2)}$$

$$\frac{\partial Q}{\partial \lambda} = \sum_{i=1}^{n} w_i (\frac{x_i}{\lambda} - d_i) = 0 \qquad \text{(S3)}$$

Solving the two equations gives the update formulas for EM algorithm, Equations (2) and (3) in the text.

Table S1. The top 50 concepts identified by Genelist Analyzer for 15 yeast genes clustered in microarray experiments.

Genes	Top 50 Concepts
Por1, Sdh1, Ndi1, Qcr7, Cox6, Rip1, Cx15, Cor1, Sdh3, Sdh4, Sdh2, Mdh1, Qcr6, Cyt1, Ach1	succinate, cytochrome, dehydrogenase, sdh, succinate dehydrogenase, sdh1, membrane, malate, mitochondria, journal, fungal, electron, mitochondrial, complex, electron transport, sdh2, transport complex, cytochrome c1, sdh3, ubiquinone, cerevisiae, heme, c1, bc1 complex, mitochondrial membrane, subunit, saccharomyce, bc 1, sdh4, complex iii, bc1, sequence, sdh4p, molecular, bc, cytochrome c, transport, sdh1b, cytochrome bc1, chemistry, b562, acetate, cor1, hap2, fv, respiratory, tcm62p, oxygen, oxidoreductase, pore

Table S2. A list of genes randomly sampled from the genome of S. cerevisiae.

Gene ID	Symbol
S000000054	Cne1
S000000367	Exo5
S000000276	Hsp26
S000002142	Pau8
S000000329	Ptc4
S000000199	Rtg3
S000007228	Scs22
S000000130	Stu1
S000000210	Uga2

Table S3. The results for the random gene list in Table S2. The significant concepts identified by Genelist Analyzer are listed here, along with their associated genes. The number in each cell indicates the number of occurrence of a concept in the document set of the corresponding gene.

Gene	chaperone	hsp gene	stress response	heat induce	thermotolerance	stress tolerance
HSP26	15	5	10	6	5	2
CNE1	2	0	0	0	0	0
UGA2	0	0	0	0	0	3
STU1	1	0	0	0	0	0
RTG3	0	0	2	0	0	0

Table S4. The results of SENT from the analysis of the yeast gene list used in Table S1. Stemming is performed for the concepts, so one word may have multiple suffixes, e.g. metabol may correspond to metabolism or metabolic.

Group	Genes	Concepts
1	Ach1	coa, acet, acetyl, acetyl coa, hydrolas, ada, dure, util, growth defect, metabol, dure growth, hydrolysi, proteom, albican, energi
2	Qcr6, Qcr7, Cyt1, Cor1	cytochrom, assembl, bound, crystal, core, membran, cytochrom oxidas, subunit, electron, respiratori chain, kda, respiratori, translocas, structur, state
3	Sdh4, Sdh2, Sdh1, Sdh3	dehydrogenas, reduct, disrupt, reduc, site, catalyt, heme, reductas, iron sulfur, product, ligand, condit, under, oxid, cycl
4	Rip1, Cox6	cytochrom oxidas, cox1, oxidas, assembl, bound, assembl factor, region, subcomplex, signal, lack, mitochondri, mrna, matur, nuclear, mtdna
5	Ndi1, Por1, Mdh1	outer, nadh, outer membran, membran, channel, mediat, life, pore, flux, mitochondria, life span, cytosol, redox, span, inhibit
